# The Sexunzipped Trial: Optimizing the Design of Online Randomized Controlled Trials

**DOI:** 10.2196/jmir.2668

**Published:** 2013-12-11

**Authors:** Julia V Bailey, Menelaos Pavlou, Andrew Copas, Ona McCarthy, Ken Carswell, Greta Rait, Graham Hart, Irwin Nazareth, Caroline Free, Rebecca French, Elizabeth Murray

**Affiliations:** ^1^e-Health UnitResearch Department of Primary Care and Population HealthUniversity College LondonLondonUnited Kingdom; ^2^Department of Statistical ScienceUniversity College LondonLondonUnited Kingdom; ^3^Department of Infection and Population HealthUniversity College LondonLondonUnited Kingdom; ^4^London School of Hygiene and Tropical MedicineLondonUnited Kingdom; ^5^Queen MaryUniversity of LondonLondonUnited Kingdom; ^6^PRIMENT Clinical Trials UnitResearch Department of Primary Care and Population HealthUniversity College LondonLondonUnited Kingdom; ^7^Faculty of Population Health SciencesUniversity College LondonLondonUnited Kingdom

**Keywords:** Internet, randomized controlled trials as topic, outcome assessment (health care), sexual health, sexually transmitted diseases, behavioral research

## Abstract

**Background:**

Sexual health problems such as unwanted pregnancy and sexually transmitted infection are important public health concerns and there is huge potential for health promotion using digital interventions. Evaluations of digital interventions are increasingly conducted online. Trial administration and data collection online offers many advantages, but concerns remain over fraudulent registration to obtain compensation, the quality of self-reported data, and high attrition.

**Objective:**

This study addresses the feasibility of several dimensions of online trial design—recruitment, online consent, participant identity verification, randomization and concealment of allocation, online data collection, data quality, and retention at 3-month follow-up.

**Methods:**

Young people aged 16 to 20 years and resident in the United Kingdom were recruited to the “Sexunzipped” online trial between November 2010 and March 2011 (n=2036). Participants filled in baseline demographic and sexual health questionnaires online and were randomized to the Sexunzipped interactive intervention website or to an information-only control website. Participants were also randomly allocated to a postal request (or no request) for a urine sample for genital chlamydia testing and receipt of a lower (£10/US$16) or higher (£20/US$32) value shopping voucher compensation for 3-month outcome data.

**Results:**

The majority of the 2006 valid participants (90.98%, 1825/2006) were aged between 18 and 20 years at enrolment, from all four countries in the United Kingdom. Most were white (89.98%, 1805/2006), most were in school or training (77.48%, 1545/1994), and 62.81% (1260/2006) of the sample were female. In total, 3.88% (79/2036) of registrations appeared to be invalid and another 4.00% (81/2006) of participants gave inconsistent responses within the questionnaire. The higher value compensation (£20/US$32) increased response rates by 6-10%, boosting retention at 3 months to 77.2% (166/215) for submission of online self-reported sexual health outcomes and 47.4% (118/249) for return of chlamydia urine samples by post.

**Conclusions:**

It was quick and efficient to recruit young people to this online trial. Our procedures for obtaining online consent, verifying participant identity, automated randomization, and concealment of allocation worked well. The optimal response rate for the online sexual health outcome measurement was comparable to face-to-face trials. Multiple methods of participant contact, requesting online data only, and higher value compensation increased trial retention at 3-month follow-up.

**Trial Registration:**

International Standard Randomized Controlled Trial Number (ISRCTN): 55651027; http://www.controlled-trials.com/ISRCTN55651027 (Archived by WebCite at http://www.webcitation.org/6LbkxdPKf).

## Introduction

Sexually transmitted infection (STI), unwanted pregnancy, and abuse within relationships are public health problems that have a high impact on young people [1,2]. There are high social and economic costs, making it important to identify cost-effective interventions [3]. Digital media interventions for sexual health have great potential because of the reach and popularity of technology such as the Internet and mobile phones, especially with young people [4]. Such interventions offer advantages over face-to-face interventions since they can be accessed privately and at users’ convenience [5] and programs can be tailored to meet users’ needs [6].

Interactive computer-based interventions for sexual health promotion can lead to improved knowledge, self-efficacy, intention, and sexual behavior (including increased condom use and reduced numbers of partners), and reduced STI [7-9], although more evidence is needed to be certain of these effects. Online trials are increasingly being used to evaluate online interventions since they offer the advantage of ease of access to large numbers of potential participants, the facility for automated randomization, reminders, data collection, and facility for blind allocation to intervention or control [10]. There is a strong argument that interventions delivered online should also be evaluated online to maximize the trial’s external validity (generalizability) [10]. However, there can be very high loss to follow-up in online trials [10], with some studies losing two-thirds of participants or more [11-13]. There is also the challenge of verifying participant identity online (to prevent repeat registrations) [14] and potential concerns about the internal validity (trustworthiness) of online data [15].

**Figure 1:**
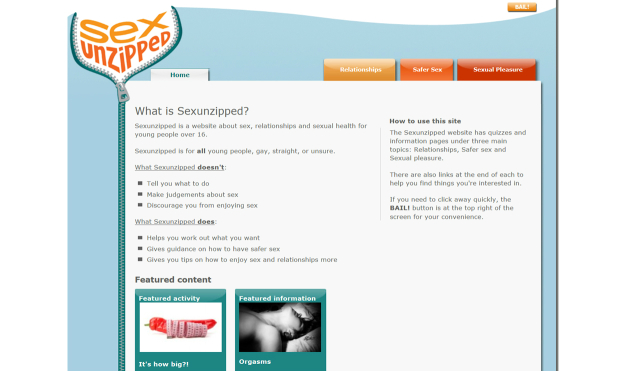
Screenshot of Sexunzipped homepage.

The “Sexunzipped” website is an interactive, theory-based website that aims to give young people the tools to make informed decisions about their sexual well-being (see Figure 1) [16-18]. We conducted a randomized controlled trial to inform the feasibility of a future definitive online randomized controlled trial to evaluate the Sexunzipped website and to contribute to knowledge about the optimal design of online trials [10] including the best ways to measure sexual health outcomes online [15].

## Methods

### Study Objectives

This study addressed the feasibility of several parameters of online trial design: recruitment route, online consent, participant identity verification, randomization procedures, concealment of allocation, online data collection, data quality, and trial retention at 3-month follow-up. Two sub-studies were conducted: (1) effect on overall response rates of asking participants to return a chlamydia urine sample by post in addition to online sexual health outcome measurement, and (2) effects on response rates of two different levels of compensation.

### Online Trial Design

Ethical committee permission was granted by the University College London ethics committee (reference 1023/002). This trial was registered with International Standard Randomized Controlled Trial Number (ISRCTN55651027).

### Participant Recruitment

We used a number of different avenues to invite young people aged 16 to 20 years to participate in the study. We emailed and telephoned staff in schools throughout the United Kingdom, asking their help to disseminate study information to pupils aged 16 years and over. Several youth organizations disseminated information, including the UK Youth Parliament and PACE (a charitable organization for lesbian, gay, bisexual, and transgender people). We also distributed printed flyers in three London sexual health clinics for young people and gave out flyers outside a large inner city school for 16 to 19 year olds. We posted an advertisement on the social networking website Facebook, making the advertisement visible only to Facebook users who were 20 years or younger and resident in the United Kingdom. Facebook imposes restrictions on sex-related advertising (including sexual health), so we could not post the advertisement to those under 18. The advertisement featured the Sexunzipped logo and asked, “Interested in sexual health? Willing to help us with our research?” Those interested clicked on the advertisement to take them to the Sexunzipped enrolment splash page. We paid a fee per click and the advertisement was withdrawn once a pre-specified daily cost limit was reached. Study information was also posted online by a number of sexual health bloggers and the study was advertised on the UK National Health Service SHO-Me website. Finally, we emailed study participants to ask them to invite friends to participate.

### Online Enrolment and Consent

Young people were invited to enroll for the research by clicking on a button on the Sexunzipped website that asked, “Are you interested in sexual health? Willing to help us with our research? Sign up for the Sexunzipped sexual health study and receive a £10 voucher for participating. Click below to find out what’s involved.” This led to two eligibility screening questions that allowed only those who said they were currently resident in the United Kingdom and aged between 16 and 20 years to register. Participants were then presented with study information and a consent form online (with checkboxes to agree or disagree with statements) and given a researcher email address and telephone number for queries. Participants were told that they (1) would be asked to complete an online sexual health questionnaire at baseline and in three months’ time, (2) would be allocated to one of two versions of a sexual health website, and (3) might also be asked to provide a urine sample for chlamydia testing to return by post. Those consenting to the research created a username and password and were then directed to an online questionnaire that elicited demographic information and measured baseline sexual health outcomes.

### Compensation Offered

Participants were offered a £10 (US$16) shopping voucher for complete follow-up data (either online questionnaire only or both online questionnaire and chlamydia urine sample). We opted for a voucher sent by post in order to ensure that participants gave valid addresses and to reduce the risk of repeat registrations in order to get the incentive. Participants allocated to receive a chlamydia test kit were sent £5 (US$8) of the compensation in advance, enclosed with the chlamydia kit. The voucher could be redeemed in a variety of different stores including clothes shops, news agents, bookshops, etc. During collection of 3-month follow-up data, we reviewed retention rates and decided to test whether a higher value voucher of £20 ($32) would increase retention. From this point onwards, the remaining participants were individually randomized to receive either a £10 or a £20 voucher (n=902) (Multimedia Appendix 1).

### Methods of Randomization

There were three individual one-to-one randomizations in the study. At recruitment, all participants were randomized in a factorial (2x2) design to either the intervention or control website and to receive or not receive a urine sample kit for chlamydia testing at follow-up. In addition, the final 902 participants were randomized after recruitment to a £10 or £20 voucher to complete follow-up as requested. The first two randomizations were performed using an automated computer algorithm and the third was performed off-site by random permutation of participant identifiers.

### Concealment of Allocation

Participants were automatically allocated by computer to control or intervention after submitting baseline data, with their passwords allowing access to either intervention *or* control website only. Neither participants nor researchers were aware of allocations in advance. There was no compensation offered for submitting baseline data. All participants were offered a £10 voucher for complete follow-up data (the 3-month online questionnaire plus the urine sample for chlamydia testing if allocated). Allocation to receipt of a chlamydia testing kit was disclosed in an email at 6 weeks, which revealed whether a chlamydia sample would be requested at 3-month follow-up. For those later allocated to the increased compensation of £20, this was revealed in the 3-month follow-up email. The trial manager (OM) was aware of allocations to the voucher and urine sample after the event, since she was responsible for postage of chlamydia test kits and appropriate value vouchers. The trial manager was not aware of allocation to intervention or control.

### Identity Verification and Consistency Checks

Participants were asked for their email address and postal address and informed that the voucher would be sent by post. We excluded participants who gave registration information that appeared fraudulent, for example, multiple registrations using the same postal address or multiple similar names or email addresses. Possible duplicate registrations were checked by manually sorting data within an Excel file. We requested date of birth and gender at baseline and also at 3-month follow-up, on the assumption that if those facts were falsified at baseline, participants may not have recalled the falsified date or gender three months later. We checked responses for inconsistent or unlikely answers (for example, selecting the first or last response option available and inconsistent responses to questions about sexual activity and condom use).

### Online Sexual Health Outcome Measurement

Demographic information including age, gender, ethnicity, and employment was collected at baseline via an online survey instrument. We used the “Sexunzipped sexual health questionnaire” to capture sexual health outcomes at baseline and again at 3-month follow-up. The Sexunzipped questionnaire contained items from available validated sexual health outcome measurement instruments including indicators for AIDS prevention programs [19], the National Survey of Sexual Attitudes and Lifestyles [20,21], and the HARK four-question scale to assess intimate partner abuse [22]. The survey instrument measured mediators of sexual behavior change (sexual health knowledge, self-efficacy, and safer sex intentions) as well as sexual behavior (condom and contraception use, use of services, and partner numbers), self-reported sexually transmitted infections, pregnancy, sexual problems, partner abuse, regretted sex, sexual pleasure, relationship satisfaction, and sexual satisfaction (Multimedia Appendix 2). All questions required mandatory responses except for questions on sexual practices. A “not applicable” option was available for the majority of the sexual health questions.

### Intervention and Control Websites

Participants were given unlimited access to their allocated website during the course of the trial. An automated email was sent to participants at 6 weeks and 9 weeks after registration to encourage exploration of the website. There was no compensation offered for engagement with the intervention or control websites.

Website usage (individual page views) was tracked through Google analytics and using bespoke (custom) software to track page views by participant unique identifier (on log-in to the website with a self-chosen username and password). We did not record time spent on the allocated websites. We chose not to track Internet Protocol (IP) addresses, since users may have received IPs that were dynamically assigned to them by Internet service providers, so they would have been liable to change.

The Sexunzipped intervention website focused on safer sex, relationships, and sexual pleasure, aiming to give young people the tools to make informed decisions about their sexual health [16]. The site content and design was informed by the integrated behavioral model and other theory, addressing mediators of behavior change such as beliefs, attitudes, perceived norms, and sense of personal agency as well as safer sex behavior and communication skills [17]. The website was structured to encourage active engagement with material, for example, quizzes that gave tailored feedback and activities that invited participants to enter personally relevant data and to reflect on decisions.

The trial control condition was an information-only control website that shared the same logo and colors as the Sexunzipped intervention site, but had no interactive activities. The website gave brief information on topics such as sexually transmitted infections, contraception, and sexual practices, but did not encourage self-reflection, decision-making, or the development of communication skills.

### Outcome Data Collection and Compensation Offered

Participants were sent an email at 13 weeks after registration, which provided a Web link to the 3-month online questionnaire. The questions asked at follow-up were identical to sexual health outcomes elicited at baseline. Non-responders were sent five further reminders by email or post (with the chlamydia test kit), and then sent a shortened version of the online questionnaire by post, containing 11 questions to be returned in a stamped addressed envelope in return for the voucher. We sent a final email to non-responders, which contained three key outcome questions in the body of the email instead of a Web link to the full survey. No compensation was offered for response to this final email. There were initial technical problems in submitting the questionnaire online, so the first 106 participants were sent the £10 voucher regardless of whether they had succeeded in submitting it.

Participants randomized to receive a urine sample kit by post at three months for genital chlamydia testing (n=1030) were sent a kit containing instructions, a urine sample container, and a prepaid envelope addressed to the laboratory. Non-responders received one repeat kit by post. Samples were analyzed by The Doctors Laboratory (TDL), using the Becton Dickinson BD Viper chlamydia Trachomatis Polymerase Chain Reaction DNA test. Results were sent by text, by telephone, or posted by mail according to participant preferences stated on the chlamydia test request form. Most participants chose to receive test results by text. Those with positive results were telephoned by the trial manager and were advised to seek treatment from local health services.

### Sample Size

A sample size of 1200 participants was estimated to provide 80% power to detect a difference in retention at the 5% significance level such as 45% vs 35% in retention rates between groups. Recruitment on Facebook was so straightforward and cheap that we decided to exceed this target number. However, this resulted in large numbers of participants aged 18 years and over and a much smaller proportion of younger participants (see Table 1).

**Table 1:** Participant age at enrolment (by gender, male or female).

Age in years	Female, n=1259, n (%)	Male, n=735, n (%)	Total, n=1994, n (%)
16	41 (3.26)	29 (3.95)	70 (3.51)
17	72 (5.72)	32 (4.35)	104 (5.22)
18	448 (35.58)	213 (28.98)	661 (33.15)
19	410 (32.57)	268 (36.46)	678 (34.00)
20	287 (22.80)	192 (26.12)	479 (24.02)
21 or more	1 (0.08)	1 (0.14)	2 (0.10)
Total	1259 (100)	735 (100)	1994 (100)

### Data Analysis

The primary outcome for this feasibility study was retention of valid participants at 3-month follow-up, that is, completion of the online sexual health questionnaire only or both sexual health questionnaire and chlamydia urine sample (according to prior allocations). We analyzed measures of feasibility and process including numbers recruited by source, number of rejected ID verifications, numbers responding to email and postal follow-up prompts by level of compensation and allocation to chlamydia test kit, and proportion of urine samples testing positive for genital chlamydia. For the entire group of participants, the probability of retention was modeled in terms of group allocation, website usage, level of compensation, demographic variables, and sexual behavior determinants, using univariate and multivariate logistic regression. Retention was defined as response to requests for follow-up data (online or postal questionnaire and chlamydia test sample) at 3 months. In univariate analysis, we explored how each variable was individually associated with retention (see Multimedia Appendix 3). We subsequently performed multivariate logistic regression where all the variables were included concurrently in the regression model. Significant predictors of retention were then identified using a forward stepwise selection procedure (the significance levels for removal and addition to the model were .1 and .05, respectively) starting with all predictors in the model (see Multimedia Appendix 3). A *P* value of .05 or less was considered statistically significant. Statistical analyses were conducted using STATA Version 12.

## Results

### Online Trial Eligibility, Recruitment, and Retention

Multimedia Appendix 1 indicates the numbers eligible, recruited, excluded, and retained at follow-up. Since the outcome of interest was retention at 3 months, *all* participants (including non-responders at 3 months) were included in analyses.

### Participant Identity Verification and Data Quality

After registration, 18 participants asked to be withdrawn from the study. Of these, 7 gave a reason: 3 did not want to give a urine sample, 2 were concerned about mail coming to the house, and 2 said their friends had enrolled them as a joke. There were 12 participants who appeared to have registered more than once (on the basis of the same or very similar names, email, or postal addresses). These participants were removed, leaving 2006 participants retained in the analysis of the effect of voucher compensation.

No participants chose extremes of response option for every question (for example, selecting the first or last response option available). In total, 66 participants (3.29%, 66/2006) gave discrepant dates of birth at baseline and 3 months later, and one person wrote nonsense (unintelligible content) in many of the free-text boxes. In addition, some participants gave inconsistent answers within the baseline questionnaire: 15 participants (0.75%, 15/2006) indicated that they were in a sexual relationship but also that they had never had sex (involving genital contact), 50 participants (2.49%, 50/2006) said that they had not used a condom at last vaginal sex but also reported no episodes of unprotected vaginal sex in the last three months, and 11 participants supplied answers to questions that should have been skipped (on the basis of their previous answers). Furthermore, 9 participants reported discrepant genders at baseline and follow-up, and 2 participants were 21 or over, suggesting they must have entered an age between 16 and 20 years in response to the initial eligibility screening questions, but later submitted dates of birth out of this range in the online questionnaires.

**Figure 2:**
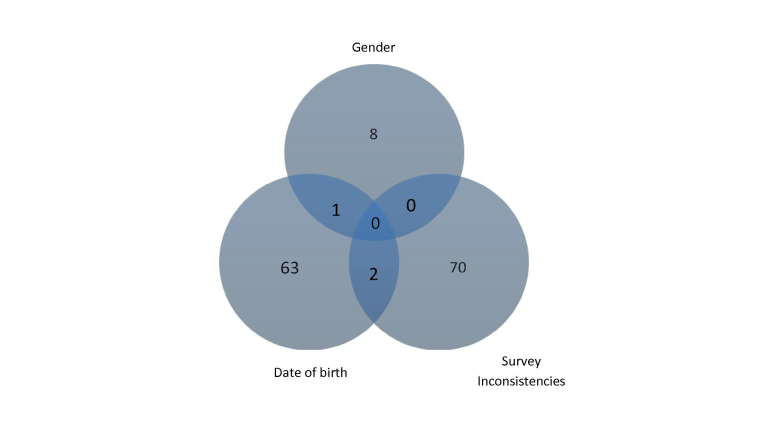
Numbers of participants giving discrepant or inconsistent responses.

It is difficult to evaluate these inconsistencies—there may have been data entry mistakes, technical problems with the questionnaire skip pattern, questions may have been interpreted in ways that we had not anticipated, and it is possible that some participants may have changed gender identity between baseline and follow-up. A total of 3.88% (79/2036) entries were therefore deemed invalid on the basis of repeat contact details, discrepant dates of birth, or nonsense responses, and a further 4.00% (81/2006) participants gave an inconsistent gender at two time points or inconsistent responses to sexual health questions. There was little overlap between these inconsistencies (see Figure 2).

Our analyses excluded participants who appeared to have registered more than once, but retained those with inconsistent responses (total n=2006), since these respondents contribute to understanding the effect of increased compensation.

### Participant Recruitment

Most of the 2006 participants were recruited via an advertisement on Facebook (84.00%, 1685/2006), with 8.97% (180/2006) via friends or relatives, 3.99% (80/2006) via email, and only 1.99% (40/2006) through school or college. There were an estimated 2,846,204 Facebook users resident in the United Kingdom who were aged 18-20 years in 2010 (47% female and 53% male) [23], but we do not know how many of the target audience actually saw the advertisement, since it was withdrawn once a daily cost limit was reached. An estimated 6705 people viewed the Sexunzipped enrolment website (figure estimated from Google analytics and bespoke page view tracking software). Of these, 4926 met the eligibility criteria for age and UK residence, 2600 went on to submit online consent forms, 2207 created user accounts (supplying usernames and passwords), and 2036 submitted baseline demographic and sexual health data (41.33%, 2036/4926) of people meeting the eligibility criteria. After participant withdrawals and removal of those who appeared to have registered more than once, 2006 people remained (see Multimedia Appendix 1).

### Online Trial Participants

Trial participants (n=2006) were recruited between November 2010 to March 2011. Two-thirds of the sample (62.81%, 1260/2006) were female, 36.59% (734/2006) male, 0.25% (5/2006) female to male transgender, 0.99% (2/2006) male to female transgender, and 0.25% (5/2006) “other”. Participants ranged in age from 16 to 21 years, with a median age of 19 years (see Table 1, presented by gender). Participants were recruited from all four countries in the United Kingdom, with 81.40% (1633/2006) from England, 7.63% (153/2006) from Scotland, 3.84% (77/2006) from Wales, and 4.64% (93/2006) whose location could not be deduced from the postal code supplied. The majority of participants were white (89.17%, 1778/1994) (see Table 2, presented by gender). Most participants were students at school, college, or university (77.48%, 1545/1994), with 28.18% (562/1994) in employment, 8.02% (160/1994) unemployed, 2.00% (40/1994) in a “gap year” before college or university, 1.00% (20) sick or disabled, and 1.00% (20/1994) full-time parents or caregivers.

At baseline, 1229/2006 participants (61.27%) reported being in a relationship with one person, 65/2006 (3.24%) were in relationships with more than one person, and 711/2006 (35.44%) were not in a relationship. Of the latter group, 80 (3.99% of the whole sample, 80/2006) had never been in a relationship. The majority of current or past relationships were sexual (92.00%, 1772/1926) and only 108/2006 of the sample (5.38%) had never had (genital) sex. Most participants reported predominantly opposite-gender sexual attraction (87.45%, 1101/1259 of female participants and 77.14%, 567/735 of male participants) (see Table 3).

We were concerned that participants might re-register to gain access to the intervention site, but there was no evidence of this: 51.54%, 1034/2006 were allocated to the intervention site, which is consistent with random variation in allocations (95% CI 49.4-53.7). The majority of participants (75.77%, 1520/2006) visited the intervention or comparator websites, with 29.91% (600/2006) visiting 11 or more web pages (see Table 4).

**Table 2:** Ethnicity (by gender, male or female).

Ethnicity	Female, n=1259, n (%)	Male, n=735, n (%)	Total, n=1994, n (%)
White British	1051 (83.47)	628 (85.44)	1679 (84.20)
White Irish	26 (2.07)	20 (2.72)	46 (2.31)
White other	30 (2.38)	23 (3.13)	53 (2.66)
Asian British, South East Asian, Chinese	27 (2.14)	26 (3.54)	53 (2.66)
Black British, African, Caribbean	40 (3.18)	8 (1.09)	48 (2.40)
Mixed cultural background	48 (3.81)	15 (2.04)	63 (3.16)
Other background	2 (0.16)	0 (0.0)	2 (0.10)
Prefer not to say	35 (2.78)	15 (2.04)	50 (2.51)
Total	1259 (100)	735 (100)	1994 (100)

**Table 3:** Sexual attraction (by gender, male or female).

Sexual attraction	Female, n=1259, n (%)	Male, n=735, n (%)
Only to females, never to males	14 (1.11)	443 (60.27)
More often to females and at least once to males	49 (3.89)	124 (16.87)
About equally often to females and to males	88 (6.99)	32 (4.35)
More often to males and at least once to females	488 (38.76)	51 (6.94)
Only to males and never to females	613 (48.69)	81 (11.02)
I have never felt sexually attracted to anyone	7 (0.56)	4 (0.54)
Total	1259 (100)	735 (100)

**Table 4:** Number of pages of intervention or comparator websites viewed.

Number of pages viewed	Allocated to intervention website, n=1034, n (%)	Allocated to comparator website, n=972, n (%)	All participants, n=2006, n (%)
0	251 (24.27)	235 (24.18)	486 (24.23)
1-5	346 (33.46)	229 (23.56)	575 (28.66)
6-10	176 (17.02)	169 (17.39)	345 (17.20)
11 or more	261 (25.24)	339 (34.88)	600 (29.91)
Total	1034 (100)	972 (100)	2006 (100)

### Outcome Data Collection

We reported the response rates to each round of prompting for the online sexual health questionnaire (n=2006) and postal chlamydia samples (n=1030) at 3-month follow-up.

### Online Questionnaire

The overall response rate for the 3-month online sexual health outcome questionnaire was 71.78% (1440/2006), combining responses to emailed and postal follow-up invitations.

### Follow-Up Emails With Web Link to the Online Questionnaire

In total, 60.22% of participants (1208/2006) completed the online questionnaire in response to an email at 3 months with a Web link to the survey instrument: 36.09% (724/2006) responded to the first emailed request; 11.62% (233/2006) to the second reminder (by email or postal, with the chlamydia sample); 4.99% (100/2006) to the third; 3.39% (68/2006) to the fourth; and 4.09% (82/2006) to the final email reminder.

### Follow-Up by Post

Non-responders (798 participants) were sent the shortened 11-question version of the online questionnaire by post. In total, 208/798 (26.07%) responded to this postal follow-up, boosting the overall response rate by 10.37% (208/2006); 79 paper questionnaires were returned uncompleted (9.90%, 79/798), marked by the UK Royal Mail as “Addressee gone away” or “Incorrect or incomplete address or name”; and 11 people returned the questionnaire without completing it.

### Follow-Up Emails With Questions in the Email Body

We sent one final email to the 560 remaining non-responders, with three key outcome questions in the body of the email text instead of a Web link to the full online survey. Of these emails, 42/560 (7.50%) bounced back (ie, were invalid email addresses), 27 people (4.82%, 27/560) responded (1.35%, 27/2006 of the total sample), providing data on self-reported chlamydia in the last 3 months and condom use at last anal and vaginal sex.

### Postal Urine Sample for Chlamydia Testing

In total, 1030 participants were asked to return a urine sample for chlamydia testing: 32.14% (331/1030) returned the urine sample after the first postal invitation, one sample required a second posting following damage in the post, and a further 12.72% returned the urine sample after the second request (131/1030). Five additional samples were returned but could not be processed: two were mislabeled, two were insufficient samples, and the laboratory was “unable to process” one sample, giving an overall response rate of 44.95% for processed samples (463/1030). There was no response from 54.56% of participants (562/1030); of these non-responders, 15 sample kits were returned with wrong or incomplete addresses, 14 were returned “addressee unknown” or “gone away”, and 20 sample kits were returned uncollected from post offices. Of the urine samples that could be processed, 11/463 (2.38%) tested positive for chlamydia Trachomatis on a Nucleic Acid Amplification Test (NAAT).

### Impact on Response Rates of Requesting a Urine Sample by Post

Of the 976 participants who were asked only to complete the 3-month questionnaire, 736/976 (75.41%) completed it. Requesting a chlamydia test urine sample in addition to the online sexual health questionnaire reduced the retention rate considerably, with only 429 out of the 1030 (41.65%) completing both. A total of 31/1030 participants (3.01%) sent back the urine sample but did not complete the 3-month questionnaire, and 275/1030 (26.70%) completed the 3-month questionnaire but did not send back the urine sample. Being asked to return a urine sample as well as to fill in the online questionnaire significantly reduced the overall response rates for complete outcome data (41.65%, 429/1030 vs 75.41%, 736/976, *P*<.001).

### Levels of Compensation Offered

To test the effect of compensation offered, 902 participants were randomized to receive either a £10 or a £20 voucher for complete follow-up data. Table 5 shows the effect of doubling the compensation to £20 and the effect of being asked to fill in the online questionnaire and return a urine sample for chlamydia testing. A higher value voucher boosted response rates by 6-10%.

**Table 5:** Response rates at 3-month follow-up by level of compensation offered and chlamydia sample request.

Allocation (total n=902)	Asked to fill in online questionnaire only (n=417)	Asked to fill in online questionnaire and chlamydia sampling (n=485)		
	Questionnaire completion rate n (%)	Questionnaire completion rate n (%)	Chlamydia sample response rate n (%)	Complete data set (both questionnaire and chlamydia sample) n (%)
£10 voucher	144/202 (71.29)	149/236 (63.14)	97/236 (41.10)	91/236 (38.56)
£20 voucher	166/215 (77.21)	183/249 (73.49)	118/249 (47.39)	111^a^/249 (44.58)
*P* value (chi-square significance test)	.20	.01	.19	.21

^a^Includes one sample returned but not processed (insufficient sample).

### Analysis of Retention at Three Months

There was no differential retention by allocation to control or intervention group, age, living in London, recruitment route (Facebook vs other routes including email, known contacts, leaflets, or posters), being in a sexual relationship, time since last sex, safer sex (defined as condom use at last vaginal or anal sex), last sex with a regular/non-regular partner, having talked about sexual desires, sexual problems, regretted sex, vaginal or anal sex at last sex, nor by range of sexual activities (see Multimedia Appendix 3). We found significantly lower retention for males compared to females (OR 0.61, 95% CI 0.49-0.75), “non-white” participants compared to white (OR 0.58, 95% CI 0.44-0.75), participants who had ever had sex (OR 0.47, 95% CI 0.27-0.81), and allocation to receive a chlamydia test kit (OR 0.65, 95% CI 0.53-0.79), and significantly higher retention among those who were attracted only to same-gender partners (OR 1.84, 95% CI 1.28-2.65), those at school, college, university, or training (OR 1.73, 95% CI 1.37-2.20), in a relationship with one person (OR 1.38, 95% CI 1.11-1.71), and in relationships of more than one week (OR 2.65, 95% CI 1.15-6.11). Higher engagement with the control/intervention websites was associated with increased probability of retention at 3-month follow-up and, as expected from the results reported above, participants who were given the higher value voucher (£20) were more likely to submit follow-up data than participants in the £10 voucher group (OR 1.29, 95% CI 1.03-1.61).

## Discussion

### Principal Findings

The online trial methodology used to test the Sexunzipped website proved acceptable to young people, as evidenced by retention at three months, which was comparable with retention rates in a school-based longitudinal cohort study [24]. Online recruitment to the trial was quick and easy [12,13]. A low proportion (3.88%) of apparently fraudulent enrolment was detected by manually checking contact details, checking for unlikely response options or meaningless free-text responses, and requesting date of birth at baseline and follow-up. The internal validity of our online data was good, with 96.0% supplying consistent responses within the sexual health questionnaire.

Higher value compensation increased response rates by 6-10%, yielding a maximal retention rate at 3-month follow-up of 77.21% for the online questionnaire and 47.39% for the postal chlamydia sample with a £20 shopping voucher. Online sexual health outcome measurement is therefore an efficient method of gathering self-reported outcome data of good quality. Our findings align with other studies that report higher retention with repeated reminders, multiple routes of contact (including email, text message, post, and telephone), and higher value compensation [25,26].

### Recruitment and Participants

The simplest and most effective route for recruitment was an advertisement on the social networking website Facebook, displayed only to a specific target population. However, since Facebook does not permit advertising with references to sex or sexual health to be displayed to those under 18, there are proportionately fewer of the younger age groups (16-17 year olds) in our sample (Table 1). Young women were over-represented (two-thirds of the sample), despite an even distribution in the gender of young adult Facebook users [23]. Young men were less likely to participate in this study and more likely to drop out by 3 months. We would have liked to have conducted this research with young people under 16 because Sexunzipped website content is particularly appropriate for those around the age of sexual debut (which is at a median age of 16 in Britain [21]), but this would require parental consent. Recruitment via Facebook was much cheaper, quicker, and easier than other avenues of recruitment; however, restriction on advertising to those 18 and over resulted in recruitment of participants with a higher mean age than intended. Since our Facebook advertisement was displayed only to those within our target age group and living in the UK, this will have helped to limit the possibility for fraudulent enrolment by those not meeting these criteria.

### Feasibility of Online Outcome Data Collection

A particular challenge for online trials is attrition, with online studies often recruiting large numbers of participants but losing the majority by follow-up [11-13]. Our maximal retention rate for the online questionnaire (77.21%) is comparable with rates achieved in another online trial that also offered compensation and multiple follow-up reminders (79% retention at one month and 53% at two-month follow-up) [25]. This aligns with others’ findings that an online questionnaire (via a Web link in an email) produces better response rates than the same questionnaire sent by post and that offering increased compensation does have a significant impact on response rates [27]. Route of recruitment (Facebook vs other more personal routes of contact) had no effect on retention at 3 months, which supports the use of online advertising for participant recruitment.

The Sexunzipped online questionnaire was long (a maximum of 103 sexual health questions depending upon skip patterns, plus 8 questions for demographic information and contact details). Young people involved in field work for this project had previously said that they would not be willing to fill in a long questionnaire [16], but only 7.75% (171/2207) of people who created user accounts did not go on to submit baseline demographic and sexual health data. In our parallel qualitative evaluation of the trial design, young people said that they enjoyed responding to questions that they felt were relevant to them (companion paper, [28]) and our retention rates support this finding. Data sets were complete for those who submitted questionnaires online and the proportion of inconsistent responses was small (4.00%). By their nature, quantitative survey questions cannot capture subtleties of meaning for individual respondents [29]; however, our analysis of qualitative comments on the questionnaire indicated that for the most part it was judged appropriate and acceptable [28].

It is difficult to evaluate questionnaire inconsistencies since it is impossible to know whether an inconsistency represents a data entry error, misunderstanding of a question, or dishonest reporting. We removed from analysis those with repeat contact details, discrepant dates of birth, or nonsense responses, judging these to indicate possible dishonesty. Removing *all* participants with inconsistent responses would exclude those whose responses were dishonest but also those who made genuine errors on a minority of questions. This would increase the internal validity of data, but could also result in a selection bias.

### Feasibility of Chlamydia Sampling

While the Sexunzipped trial was conducted principally online, a sub-sample were asked to return chlamydia test samples by post since biological outcomes are the most reliable indicators of the impact of sexual health interventions [30]. We chose to measure Genital chlamydia infection since it is the most prevalent STI in young people [31] and a Polymerase Chain Reaction test on a urine sample is non-invasive and has high accuracy. To maximize return of the samples for chlamydia testing, we implemented many of the techniques known to increase postal response rates for questionnaires [32]: we emailed participants before dispatching test kits, provided stamped return envelopes, included clear, short, personalized covering letters and chlamydia request forms, offered half of the compensation in advance, and posted second sample kits to non-responders. A proportion of sample kits (1.46%, 15/1030) were returned because of an incorrect address and 1.94% (20/1030) were not collected from post office delivery offices; the secure “biohazard” packaging may have meant that the chlamydia samples did not fit through some household letter boxes.

Chlamydia screening is opportunistic in the United Kingdom. Most screening is done via clinics or outreach schemes, but in some areas young people are contacted by post for chlamydia screening and there are also websites through which those under 25 years of age can request a postal chlamydia testing kit. Our overall response rate of 44.66% (460/1030) for chlamydia urine samples compares favorably with postal chlamydia screening initiatives (typically about 25% return rate after two postal invitations without compensation) [33], but requesting a postal chlamydia sample as well as online questionnaire data almost halved the overall response rates at 3-month follow-up. Our qualitative evaluation suggests that those who had had a recent chlamydia test may have been less motivated to receive another test result [28]. In our data, those who reported an STI check-up within the last 3 months were less likely to return a sample (41.28%, 116/281), than those who had not had a recent check-up (46.56%, 332/713), but this difference was not statistically significant (*P*=.132).

We found a similar point prevalence of chlamydia (2.38%) to that found by the UK national chlamydia screening program, which reported 2.1% positive diagnoses in 15 to 25 year olds screened in 2011 [31]. One-quarter of our sample (24.32%, 293/1205) reported an STI check up in the last 3 months (26.13%, 208/796, of female participants and 21.14%, 85/402, of male); in comparison, the UK chlamydia screening program reached an estimated 42.7% for young women and 22.6% of young men over the entire year 2010-11. The cumulative incidence of self-reported genital chlamydia (diagnosis and/or treatment over the previous 3 months) was 6.39% (77/1205), taking data from the 3-month follow-up online questionnaire.

### Efforts to Increase Retention

We found that multiple reminders via two methods of contact (by email and by post) were acceptable to young people [28] and increased overall response rates from 36.09% (724/2006) to 71.78% (1440/2006) for the sexual health questionnaire and from 32.14% (331/1030) to 44.95% (463/1030) for the postal chlamydia test sample. We could have sought mobile phone numbers as a third avenue for contact [25]; however, contact by post and telephone is more resource intensive than automated emails. Outcome data collected via mobile phone may incur a cost for participants and there is more of a limit on the number of questions that can be asked. Young people may change email addresses and postal addresses frequently, so mechanisms are needed to keep these up to date, for example, requesting two different email addresses and using an email address to log in, which would prompt users to keep the address up to date.

A higher level of compensation increased the response rates by 6 to 10% for the postal chlamydia test sample and the online questionnaire at 3-month follow-up (Table 5), but these differences were not statistically significant for the most part. Requesting a chlamydia sample as well as the online questionnaire had an adverse impact on the response rate for the online questionnaire, but a higher level of compensation mitigated this, increasing the response rate from 63.14% (149/236) to 73.49% (183/249) (*P*=.01).

### Limitations

The sample was a convenience sample, with participants self-selecting into this trial. This means that the sample is not representative of UK youth nor of Facebook users, which limits the generalizability of the overall findings. We recruited a diverse sample in terms of geographical location and ethnicity: 10.78% of trial participants were “non-white”, which compares with a 14% non-white population in England and Wales [34] and 2% in Scotland [35].

While our best retention rate compares favorably with many other online trials, any drop-out at follow-up limits the validity of data on intervention efficacy [10]. There was no differential retention by allocation to control or intervention group, which is important in terms of assessing the impact of the intervention. However, bias may be introduced by the fact that those retained in the trial were more engaged with the intervention/control and were more likely to be female, white, attracted to the same gender, at school/college/university, to have never had sex, and to be in relationships with one person for more than a week.

We under-recruited younger participants (aged 16-17 years); while Facebook is quick and cheap, it is probably necessary to invest more resources to attract younger participants specifically, perhaps through sexual health websites. Ideally, we would have liked to recruit 13-16 year olds, but the necessity for parental consent for participation in research makes this group hard to reach. We decided that an online form for parental consent would not be adequate, since this could be completed fraudulently by participants.

The first 106 participants experienced technical problems with the submission of the questionnaire; technical problems are a constant threat to online research and can be minimized by rigorous testing before systems go live. We designed the questionnaire with skip patterns so that irrelevant questions would not be presented. Despite pre-trial questionnaire testing, a small percentage of participants (0.55%, 11/2006) supplied answers to questions that should have been skipped, which we cannot explain.

This feasibility trial was strengthened considerably by the change of protocol on compensation level at mid-point. We decided to offer the higher incentive on review of the retention data and this has allowed us to report the success of this approach. The mid-point randomizations were generated by computer and were conducted off-site, so we have no concerns about the robustness of our trial procedures.

### Future Directions

Recommendations for the conduct of online randomized controlled trials and online sexual health research can be found in Multimedia Appendix 4. These recommendations were derived from this quantitative evaluation and from the linked qualitative evaluation of the Sexunzipped trial procedures reported in a companion paper [28].

### Conclusions

There is increasing realization of the potential for digital interventions for sexual health promotion [36] and for innovative data collection methods via digital media [37]. Online evaluation offers many advantages including access to hard-to-reach populations and user-friendly, efficient, and cost-effective research administration and data collection mechanisms [15]. This paper contributes to understanding how to improve retention and ensure good quality sexual health outcome measurement in an online research environment.

## Multimedia Appendix 1

Flow diagram – recruitment, allocations, and retention.

## Multimedia Appendix 2

Sexunzipped sexual health questionnaire.

## Multimedia Appendix 3

Predictors of retention at 3-month follow-up.

## Multimedia Appendix 4

Recommendations for the conduct of online trials and sexual health research.

## Abbreviations

IP: Internet Protocol
NAAT: Nucleic Acid Amplification Test
STI: sexually transmitted infection
