# Supplementary figures and images for: The Sexunzipped Trial: Optimizing the Design of Online Randomized Controlled Trials
Source: J Med Internet Res. 2013 Dec 11;15(12):e278. doi: 10.2196/jmir.2668 (PMC3868980; doi:10.2196/jmir.2668)

**Figure 1 - Flow Diagram – Recruitment, allocations and retention**

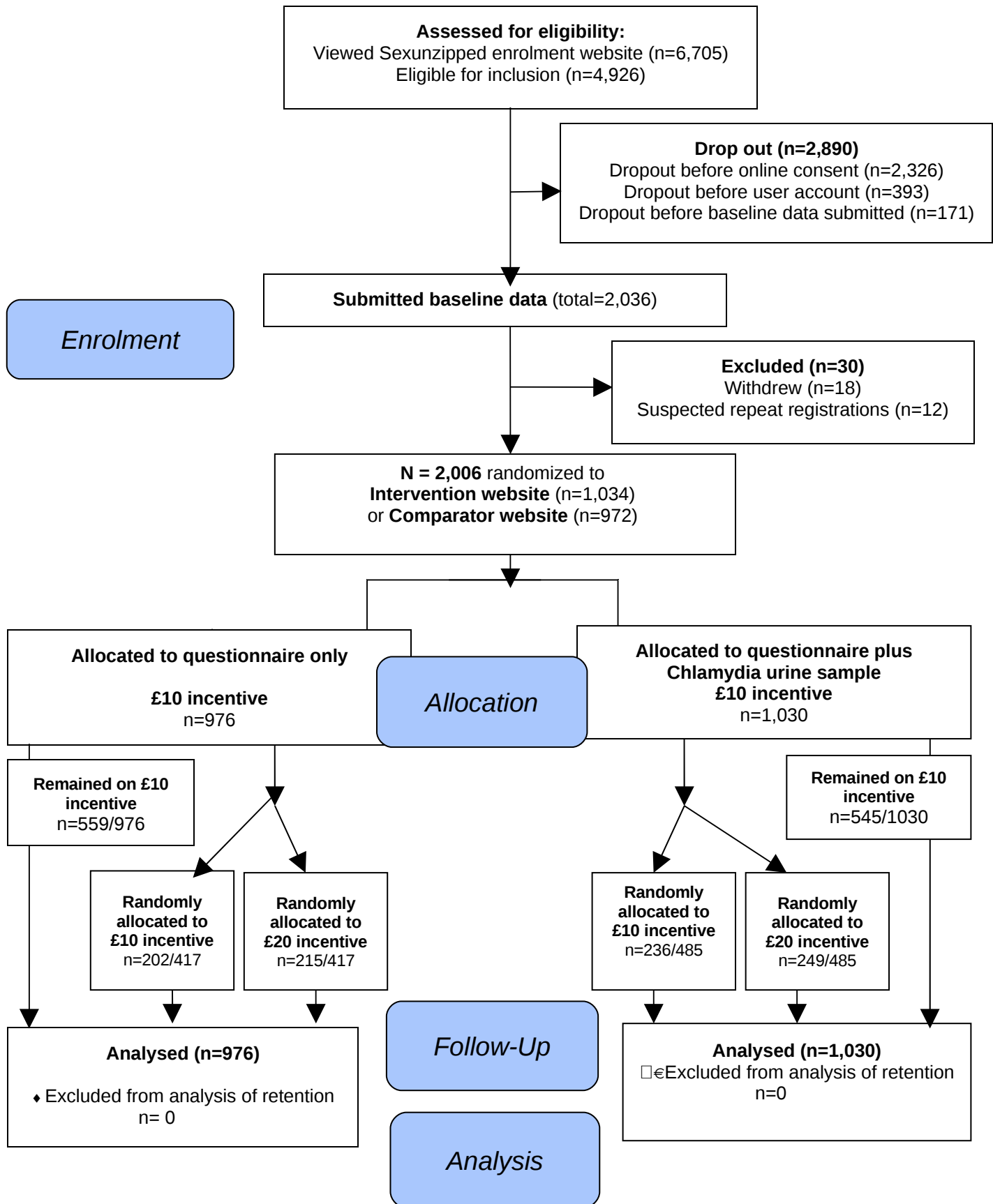

Supplement: Supplementary file 1 [file jmir_v15i12e278_app1.pdf]
