# Supplementary material for: The Sexunzipped Trial: Optimizing the Design of Online Randomized Controlled Trials
Source: J Med Internet Res. 2013 Dec 11;15(12):e278. doi: 10.2196/jmir.2668 (PMC3868980; doi:10.2196/jmir.2668)
Supplement: Supplementary file 3 [file jmir_v15i12e278_app3.pdf]

	Retention		Unadjusted Odds Ratio			Fully adjusted Odds Ratio (after stepwise selection)		
**Factor**	**No**	**%**	**O.R.**	**95% C.I.**	**P-value**	**O.R**	**95% C.I.**	**P-value**
**Age**								
16-17	51	73	1	-	-			
18 or more	1388	70	0.87	(0.51, 1.48)	0.601			
**Gender**								
Female	922	74	1	-	-	1	-	-
Male	467	64	0.63	(0.52, 0.77)	**<0.001**	0.61	(0.49, 0.75)	<0.001
**Ethnicity**								
White	1197	72	1	-	-	1	-	-
Non-white	192	62	0.64	(0.50, 0.82)	**<0.001**	0.58	(0.44, 0.75	<0.001
**Sexual attraction**								
Opposite gender/both genders	1241	69	1	-	-	1	-	-
Same gender attraction	148	76	1.39	(0.98, 1.95)	0.061	1.84	(1.28, 2.65)	0.001
**Incentive**								
£10	922	69	1	-	-	1	-	-
£20	467	73	1.25	(1.02, 1.55)	**0.035**	1.29	(1.03, 1.61)	0.021
**Chlamydia Kit**								
No	716	74	1	-	-	1	-	-
Yes	673	66	0.67	(0.55, 0.82)	**<0.001**	0.65	(0.53, 0.79)	<0.001
**Group**								
Control	671	70	1	-	-			
Intervention	718	70	1.03	(0.85, 1.25	0.729			
**Residence in London**								
No	1265	70	1	-	-			
Yes	124	68	0.89	(0.64, 1.23)	0.479			
**Recruited from Facebook**								
Yes	1164	70	1	-	-			
No	225	70	1.02	(0.78, 1.32)	0.909			
**In education or training**								
No	247	60	1	-	-	1	-	-
Yes	1142	73	1.76	(1.41, 2.21)	**<0.001**	1.73	(1.37, 2.20)	<0.001
**Relationship now**								
No	463	66	1	-	-	1	-	-
Yes, 1 person	888	73	1.33	(1.09, 1.63)	-	1.38	(1.11, 1.71)	-
Yes, more than 1 person	38	61	0.8	(0.47, 1.37)	**0.006**	0.71	(0.40, 1.26)	0.041
**Type of relationship** [a]								
Not sexual	96	69	1	-	-			
Sexual	1240	70	1.01	(0.69, 1.47)	0.956			

**Ever had sex**								
No	83	82	1	-	-	1	-	-
Yes	1306	69	0.49	(0.29, 0.83)	**0.007**	0.47	(0.27, 0.81)	0.007
**When had last sex** [(b)]								
Never	83	82	-	-	-			
Less than a week ago	777	71	1	-	-			
One to four weeks ago	307	66	0.81	(0.64, 1.02)	-			
One to three months ago	128	69	0.91	(0.65, 1.27)	-			
More than three months ago	94	70	0.94	(0.64, 1.39)	0.341			
**Condom use at last vaginal or anal sex** [(b)]								
No	849	71	1	-	-			
Yes	457	68	0.87	(0.71, 1.07)	0.182			
**Last partner regular** [(b)]								
No	220	66	1	-	-			
Yes	1086	70	1.21	(0.95, 1.57)	0.124			
**Relationship length** [(b)] [(c)]								
Less than one week	12	48	1	-	-	1	-	-
More than one week	1377	70	2.59	(1.17, 5.72)	**0.019**	2.65	(1.15, 6.11)	0.022
**Ever talked about sexual desires**								
No	230	74	1	-	-			
Talked at least once	1159	69	0.77	(0.59, 1.02)	0.067			
**Sexual Problem**								
No sexual problems	206	67	1	-	-			
Yes to at least one question	1183	71	1.16	(0.90, 1.51)	0.258			
**Avoided sex because of a sexual problem**								
No	889	71	1	-	-			
Yes	294	68	0.86	(0.68, 1.10)	0.219			
**Regretted sex in the last 3 months**								
No	956	71						
Regretted at least once	433	69	0.9	(0.73, 1.10)	0.308			
**Vaginal sex at last sex** [(b)]								
No	206	73	1	-	-			
Yes	1100	69	0.79	(0.60, 1.06)	0.123			
**Anal sex at last sex** [(b)]								
No	1137	68	1	-	-			
Yes	169	73	1.25	(0.92, 1.71	0.152			
**Website page-views**	-	-	1.04	(1.03, 1.05)	**<0.001**	1.04	(1.03, 1.05)	<0.001
**No of sexual activities**	-	-	0.97	(0.93, 1.01	0.07			

a) if currently in a relationship
b) if ever had genital sex
c) if last partner regular
