# Supplementary material for: The Sexunzipped Trial: Optimizing the Design of Online Randomized Controlled Trials
Source: J Med Internet Res. 2013 Dec 11;15(12):e278. doi: 10.2196/jmir.2668 (PMC3868980; doi:10.2196/jmir.2668)
Supplement: Supplementary file 4 [file jmir_v15i12e278_app4.pdf]

Recommendations for the conduct of online trials

Recruitment
* Online recruitment is cheap and highly effective (but will recruit a self-selected sample)
* Advertising via Facebook can recruit participants from pre-specified demographic groups
* Online trial procedures (registration, consent, randomization, emailed reminders and communication with researchers) are acceptable to young people and can minimize researcher work if automated
* The legitimacy of a research institution should be made clear – for example, a university logo may convey reassurance about the motivations for the study and use of data
* Repeated registrations can be minimized by collecting personal details (name, address, email address, date of birth at two time points)
* Sending compensation by post facilitates the submission of accurate postal addresses, potentially reducing fraud

Data collection
* Online data collection is acceptable and convenient for young people
* Online data collection is efficient for researchers, yielding data in forms convenient for analysis
* Online data collection can yield data with high internal consistency
* The creation of test participants who register before the main participants can provide early warning of potential problems

Follow up
* Higher value compensation increases response rates (i.e. retention at 3-month follow-up)
* Repeated email prompting increases overall response rates
* Postal follow up of non-responders boosts response rates
* Contact by telephone or text message could also be considered
* Mechanisms to keep contact details up to date are needed
* Offline contact with participants may reduce the potential for fraud, for example, compensation sent by post to guard against repeat registrations

Implications for online *sexual health* research

Recruitment
* An online environment for sexual health research is popular with young people and highly suitable for sexual health research
* It is challenging recruiting young people under 18 into online sexual health research, particularly those under 16
* Compensation encourages participation

Data collection
* Online questionnaires are convenient and acceptable to young people
* Detailed sexual health questioning online is acceptable to young people, with good completion rates and good internal validity
* Sexual health question response options should be as inclusive and non-judgmental as possible to encourage participation and honest responses
* Questions should be direct and provide clear definitions, for example, defining what is meant by 'sex' for particular questions; specifying the context for an answer – e.g. whether physical violence is unwanted or by mutual consent (as in the case of Bondage, Domination, Sadism and Masochism (BDSM) practices)
* Measurement of sexual health outcomes may prompt reflection on behavior– researchers could consider minimal baseline measurement to minimize measurement reactivity

* Research materials sent by post should be in plain packaging and not display any sexual health branding, and participants should be informed of this when enrolling
* Chlamydia urine sampling (by post) is not a good biological outcome measure in the context of poor response rates and low point prevalence
* Researchers should make clear the importance of each individual’s response for the success of the research
